# Nanocarbon from Rocket Fuel Waste: The Case of Furfuryl Alcohol-Fuming Nitric Acid Hypergolic Pair

**DOI:** 10.3390/nano11010001

**Published:** 2020-12-22

**Authors:** Nikolaos Chalmpes, Athanasios B. Bourlinos, Smita Talande, Aristides Bakandritsos, Dimitrios Moschovas, Apostolos Avgeropoulos, Michael A. Karakassides, Dimitrios Gournis

**Affiliations:** 1 Department of Materials Science & Engineering, University of Ioannina, 45110 Ioannina, Greece; chalmpesnikos@gmail.com (N.C.); dmoschov@uoi.gr (D.M.); aavger@uoi.gr (A.A.); mkarakas@uoi.gr (M.A.K.); 2 Physics Department, University of Ioannina, 45110 Ioannina, Greece; 3 Regional Centre of Advanced Technologies and Materials, Faculty of Science, Palacky University in Olomouc, Slechtitelu 27, 779 00 Olomouc, Czech Republic; smita.talande01@upol.cz (S.T.); aristeidis.bakandritsos@upol.cz (A.B.); 4 Department of Experimental Physics, Faculty of Science, Palacký University, 17. listopadu 1192/12, 779 00 Olomouc, Czech Republic

**Keywords:** nanocarbon, rocket fuels, furfuryl alcohol, fuming nitric acid, waste, hypergolics, carbon materials

## Abstract

In hypergolics two substances ignite spontaneously upon contact without external aid. Although the concept mostly applies to rocket fuels and propellants, it is only recently that hypergolics has been recognized from our group as a radically new methodology towards carbon materials synthesis. Comparatively to other preparative methods, hypergolics allows the rapid and spontaneous formation of carbon at ambient conditions in an exothermic manner (e.g., the method releases both carbon and energy at room temperature and atmospheric pressure). In an effort to further build upon the idea of hypergolic synthesis, herein we exploit a classic liquid rocket bipropellant composed of furfuryl alcohol and fuming nitric acid to prepare carbon nanosheets by simply mixing the two reagents at ambient conditions. Furfuryl alcohol served as the carbon source while fuming nitric acid as a strong oxidizer. On ignition the temperature is raised high enough to induce carbonization in a sort of in-situ pyrolytic process. Simultaneously, the released energy was directly converted into useful work, such as heating a liquid to boiling or placing Crookes radiometer into motion. Apart from its value as a new synthesis approach in materials science, carbon from rocket fuel additionally provides a practical way in processing rocket fuel waste or disposed rocket fuels.

## 1. Introduction

Furfuryl alcohol is considered an important green chemical that is mainly derived from plant raw material (corncobs, agricultural waste) through simple reaction cascades [1,2]. A notable use of furfuryl alcohol other than in organic chemistry or polymers also pertains to hypergolic rocket propellants and fuels [3,4,5,6]. In this latter case, the renewable alcohol (organic fuel) and fuming nitric acid (powerful oxidizer) react immediately and energetically upon contact (i.e., hypergolically) to release heat and gases that promote rocket lift-off (WAC Corporal, Nike Ajax). By also considering the vegetable origin of the organic compound, furfuryl alcohol fuel rocketry has been aptly highlighted in science blogs under imaginative headlines, such as “Flying to the Mars on Corncobs” (see for instance the link: https://dalinyebo.com/furfuryl-alcohol-rocket-fuel-then-and-now/). It is, however, surprising that although furfuryl alcohol is a versatile carbon precursor by pyrolysis [7,8,9,10,11,12,13,14], there is no report in the literature which acknowledges or refers in any way to the carbon residue obtained from the particular hypergolic pair. 

Recently our group has introduced hypergolic reactions as a new and general synthesis tool towards the formation of a variety of functional carbon materials (nanosheets, crystalline graphite, carbon dots, fullerols, hollow spheres, and nanodiscs) [15,16,17,18,19,20]. Hypergolic synthesis presents certain advantages over conventional carbonization methods (pyrolysis, hydrothermal, chemical vapor deposition—CVD) for two main reasons. First, the method is fast and spontaneous at ambient conditions: Carbon formation takes place by simply mixing the organic fuel and strong oxidizer at room temperature and atmospheric pressure, completing in very short time (e.g., rapid product formation). Second, hypergolic reactions are by definition exothermic and therefore release enough heat upon contact of the reagents (i.e., an energy-liberating process). This clearly differentiates from pyrolytic, hydrothermal, or CVD methods that require higher temperature in order to operate synthesis and extract carbon from its precursors (i.e., an energy-consuming process). In fact, we have previously demonstrated that the energy released from hypergolic reactions could be directly converted into useful chemical, thermoelectric, or photovoltaic work [15,17,18]. Therefore, hypergolics allows a fast and spontaneous carbon synthesis at ambient conditions in an energy-liberating manner without external stimuli (heat, spark, or mechanical shock). Nevertheless, scaling-up the method under safe conditions still remains a major challenge from a technical point of view. It is likely, though, that modern advances in rocket fuel engineering may hold the key towards the development of pilot apparatus for large-scale production in the future. Likewise, it was only the course of certain technical advances in flame-spray pyrolysis—another harsh synthetic approach towards nanomaterials—that finally allowed its safe use in labs or industry.

Under this light, the furfuryl alcohol-HNO_3_ pair certainly drew our attention as a potentially new example towards the hypergolic synthesis of carbon (note that furfuryl alcohol is considered a cheap, non-petrol derived biofuel used in fuel rocketry). Accordingly, herein we demonstrate the fast and spontaneous formation of carbon nanosheets by the ignition of furfuryl alcohol with fuming nitric acid at ambient conditions. In this case furfuryl alcohol served as the carbon source while fuming nitric acid as strong oxidizer. At the same time, we provide simple ways of converting the released energy into useful work, as for instance to boil acetone or set into motion the Crookes radiometer. The present work adds on top of previous ones from our group in this context, aiming to further highlight the wider applicability and perspective of hypergolics in carbon materials synthesis. From a materials science viewpoint, it also provides a practical way in dealing with rocket fuel waste or rocket fuel disposal. This is important taking into consideration that there is an increasing need for disposing aged stored and corrosive liquid rocket fuels in a useful manner (e.g., conversion to fertilizers or feedstock material for chemical industry; see https://www.osce.org/files/f/documents/8/f/35905.pdf).

## 2. Materials and Methods 

**Figure 1:**
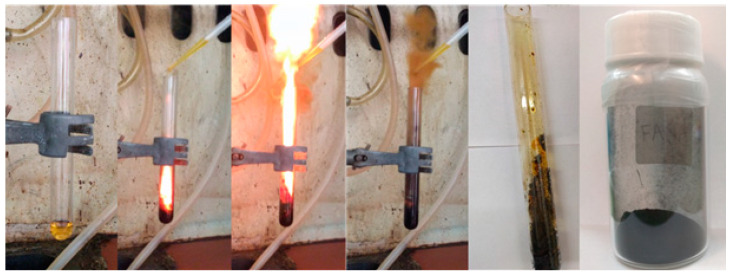
Fuming nitric acid and furfuryl alcohol reacted energetically upon contact to form a crude carbon residue inside the “rocket” test tube. Collection and washing of the residue afforded a fine carbon powder. Notice the release of brown nitrogen oxide gases as a result of nitric acid decomposition by furfuryl alcohol.

Synthesis was conducted in a fume hood with ceramic tile bench. A glass test tube (diameter: 1.6 cm; length: 16 cm) was charged with 1 mL furfuryl alcohol (99% Sigma-Aldrich, St. Louis, MO, USA) followed by the dropwise addition of 1 mL fuming nitric acid (100% Sigma-Aldrich, St. Louis, MO, USA). Both reagents reacted energetically upon contact to form a crude carbon residue inside the tube. The residue was collected and copiously washed successively with water, ethanol, and acetone prior to drying at 80 °C for 24 h. The solid was pulverized with a mortar and pestle and then sieved through a copper mesh (No. 150) to obtain a fine black powder at yield 5% (N_2_ BET specific surface area = 10 m^2^/g). The carbon yield is low compared to classic carbonization methods. This mainly has to do with the carbon precursor used in a hypergolic reaction. The discovery of new hypergolic pairs that will provide even higher carbon yields remains a challenge. The hypergolic synthesis is visualized in Figure 1. For safety reasons, synthesis was performed using small number of reagents. Accordingly, reactions were repeatedly run in multiple test tubes in order to collect enough material for characterizations (*ca.* 0.5 g). 

Powder X-ray diffraction (XRD) was performed using background-free Si wafers and Cu Kα radiation from a Bruker Advance D8 diffractometer (Bruker, Billerica, MA, USA). Raman spectra were obtained on a DXR Raman microscope using a laser excitation line at 455 nm, 2 mW (Thermo Scientific, Waltham, MA, USA). Raman measurement was conducted in bulk material using aluminum mirror as the substrate. Optical spectroscopy was conducted with a UV/Vis spectrophotometer SPECORD S 600 (Analytik Jena GmbH, Jena, Germany) operated by WinASPECT software (Analytik Jena GmbH, Jena, Germany). For the UV-Vis, the carbon solid (0.1 mg mL^−1^) was dispersed in ethanol (≥99.5% Sigma-Aldrich, St. Louis, MO, USA) and sonicated for 15 min prior to measurement. Attenuated total reflection infrared spectrum (ATR-IR) was recorded on a Thermo Nicolet iS5 FTIR spectrometer, using the Smart Orbit ZnSe ATR accessory (Thermo Fisher Scientific, Waltham, MA, USA). X-ray photoelectron spectroscopy (XPS) was carried out with a PHI VersaProbe II (Physical Electronics, Chanhassen, MN, USA) spectrometer using an Al-Kα source (15 kV, 50 W). The obtained data were evaluated with the MultiPak software package (Ulvac-PHI Inc., Miami, FL, USA). Scanning electron microscopy (SEM) images were obtained using a JEOL JSM-6510 LV SEM Microscope (JEOL Ltd., Tokyo, Japan) equipped with an X-Act EDS-detector by Oxford Instruments, Abingdon, Oxfordshire, UK (10 kV). Transmission electron microscopy (TEM) analysis was done on JEOL JEM 2100 at 200 kV (JEOL Ltd., Tokyo, Japan) using a LaB6 type emission gun (JEOL Ltd., Tokyo, Japan). Atomic force microscopy (AFM) images were collected in tapping mode with a Bruker Multimode 3D Nanoscope (Ted Pella Inc., Redding, CA, USA) using a microfabricated silicon cantilever type TAP-300G, with a tip radius of <10 nm and a force constant of approximately 20–75 N m^−1^. The Si wafers (P/Bor, single-side polished, Si-Mat) used in AFM imaging, were cleaned before use for 15 min in an ultrasonic bath (160 W) with water, acetone (≥99.5 Sigma-Aldrich, St. Louis, MO, USA), and ethanol (≥99.5 Sigma-Aldrich, St. Louis, MO, USA). All reagents were of analytical grade and used without further purification. The carbon solid was sonicated for 20 min prior AFM measurement.

## 3. Results and Discussion

**Figure 2:**
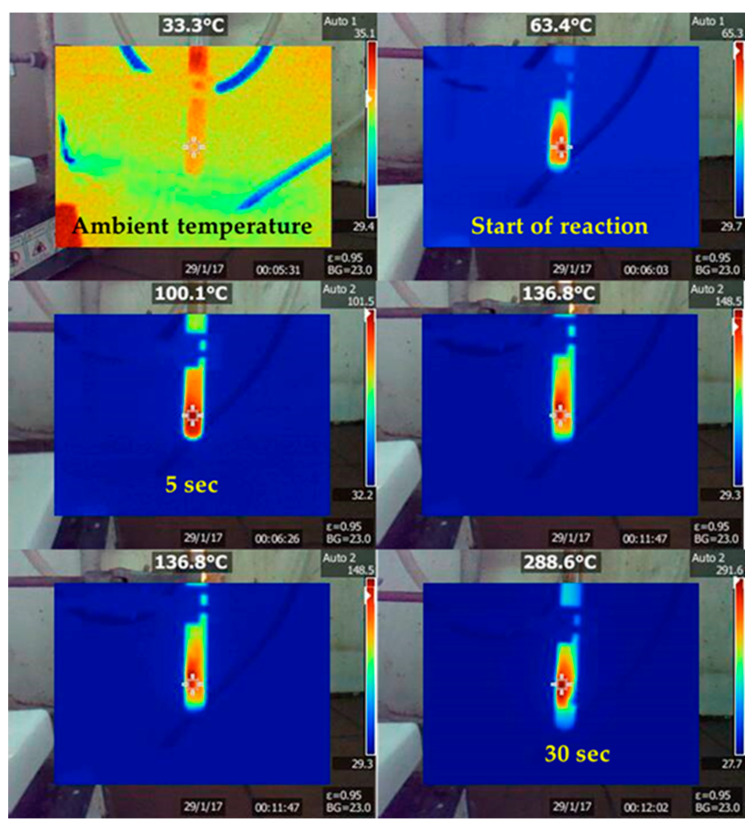
Temperature profile of the hypergolic reaction over time as depicted with a digital thermal camera. The temperature raised progressively from ambient to a maximum of nearly 300 °C within 30 s.

The hypergolic reaction of furfuryl alcohol with fuming nitric acid resulted in carbon mainly in two steps. In the first one, the alcohol underwent acid-catalyzed polymerization towards the formation of poly(furfuryl alcohol) [21]. In the second one, the energy released from the hypergolic pair provided the necessary heat and temperature needed for the in-situ carbonization of poly(furfuryl alcohol). Indeed, the temperature during hypergolic ignition reached a maximum of nearly 300 °C within 30 s, based on the thermal camera images shown in Figure 2. This temperature matched well the decomposition point of the polymer, the latter falling in the range 200–300 °C [22,23]. Any unreacted species or by-products were completely removed by thorough washing of the product with water, ethanol, and acetone, all being excellent solvents for furfuryl-based compounds. Thus, the relatively low carbon yield probably stems from the fact that the temperature of the hypergolic reaction marginally approaches the decomposition point of the polyfurfuryl alcohol intermediate (i.e., higher temperatures would normally result in higher yields).

**Figure 3:**
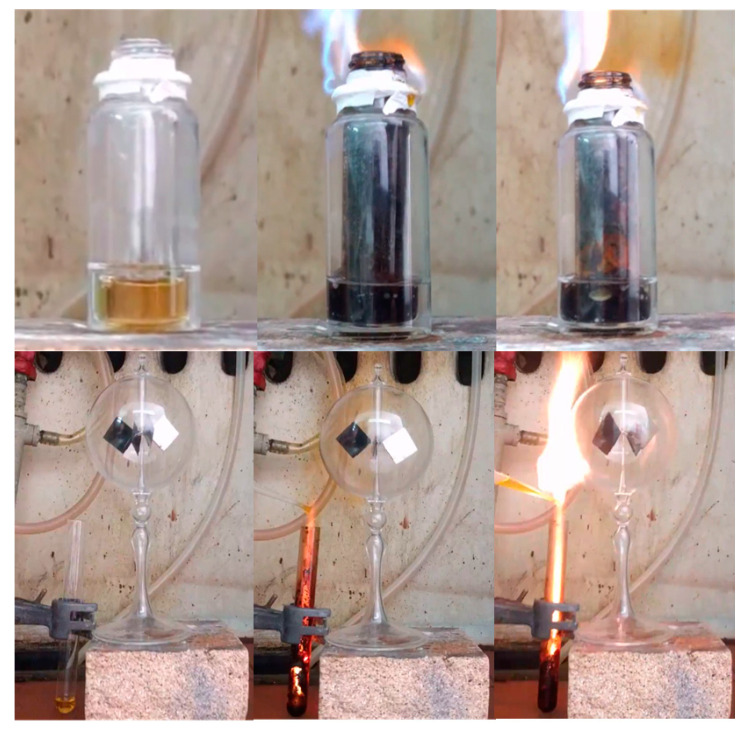
**Top**: A small glass vial filled with furfuryl alcohol was firmly fitted inside a bigger one containing acetone, as shown in the setup above. Acetone in the outer vial did not come into direct contact with the furfuryl alcohol in the inner vial. Teflon tape was placed at the contact of the fitted vials near the top rim in order to prevent flammable acetone vapor from escaping. The hypergolic reaction between furfuryl alcohol and fuming nitric acid released enough heat to boil acetone (boiling point 56 °C). The middle photo shows small bubbles that grew larger upon gradual heating of acetone to boiling (right photo). **Bottom**: A Crookes radiometer, initially at rest, was placed into motion by the released heat (e.g., notice how the polished side of the depicted vane changed position upon rotation). Spinning was relatively slow due to non-uniform heating of the radiometer.

The heat released from the reaction was exploited in various ways to produce useful work. In a first example, it was possible to boil acetone without using a heating device (Figure 3, top). This example was inspired by the popular chemistry demonstration experiment of boiling acetone through the exothermic dissolution of sulfuric acid in water. Worth noting, the operation of boiler heaters very much depends on similar energy transfer from an exothermic reaction, such as oil combustion, to a fluid, such as water. In a second example, the reaction heat was directly converted into mechanical motion through a heat engine (e.g., Crookes radiometer) (Figure 3, bottom). These simple examples do not represent actual applications but rather serve as educational displays on chemical energy conversion.

Furthermore, they show some additional ways of exploiting the released energy from hypergolic reactions that are complementary to the chemical, thermoelectric, or photovoltaic work presented elsewhere [15,17,18]. Hence hypergolics not only enables an operationally simple carbon synthesis but also gives off useful energy in the process. 

**Figure 4:**
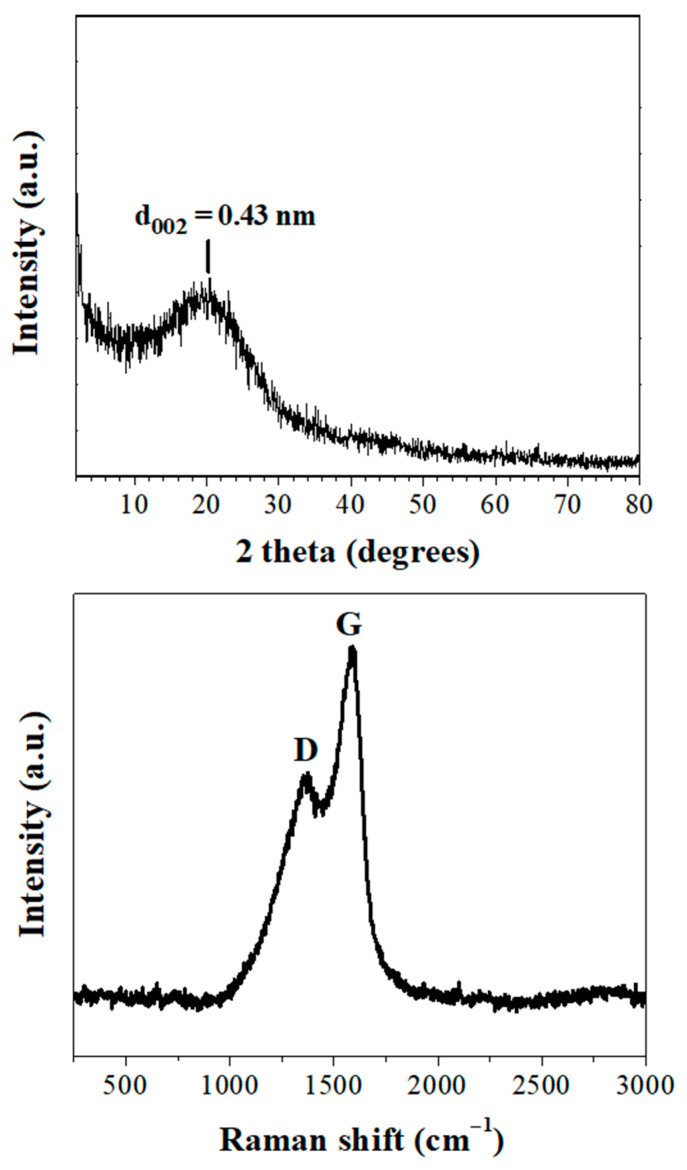
X-ray diffraction (XRD) pattern (**top**) and Raman spectrum (**bottom**) of carbon nanosheets.

X-ray diffraction and Raman spectroscopy were used in combination to unequivocally identify carbon formation [24,25]. The XRD pattern (Figure 4, top) showed a broad (002) reflection associated with graphite. The interlayer spacing (d_002_ = 0.43 nm) was considerably higher than that of graphite (d_002_ = 0.34 nm), thus indicating the formation of turbostratic carbon (e.g., structural ordering in between that of amorphous carbon and crystalline graphite). In general, the d_002_ value is often used to estimate the graphitization degree of carbon: the larger the d_002_ value, the larger the lattice disorder will be. Raman spectroscopy displayed the characteristic G and D bands for graphitic materials [26] at 1591 cm^−1^ and 1358 cm^−1^, respectively (Figure 4, bottom). The G band is usually associated with graphitic sp^2^ carbons while the D band with sp^3^ carbons. Graphitic domains are thermodynamically favored at ambient conditions, whereas the sp^3^ domains probably result from the insertion of oxygen- and nitrogen-containing functionalities in the carbon lattice (*vide infra*). In spite of the fact that sp^2^ carbons prevailed, the relative intensity ratio I_D_/I_G_ = 0.6 was still significantly higher than the value of 0.1–0.2 for crystalline graphite [16], thus confirming a disordered carbon structure.

**Figure 5:**
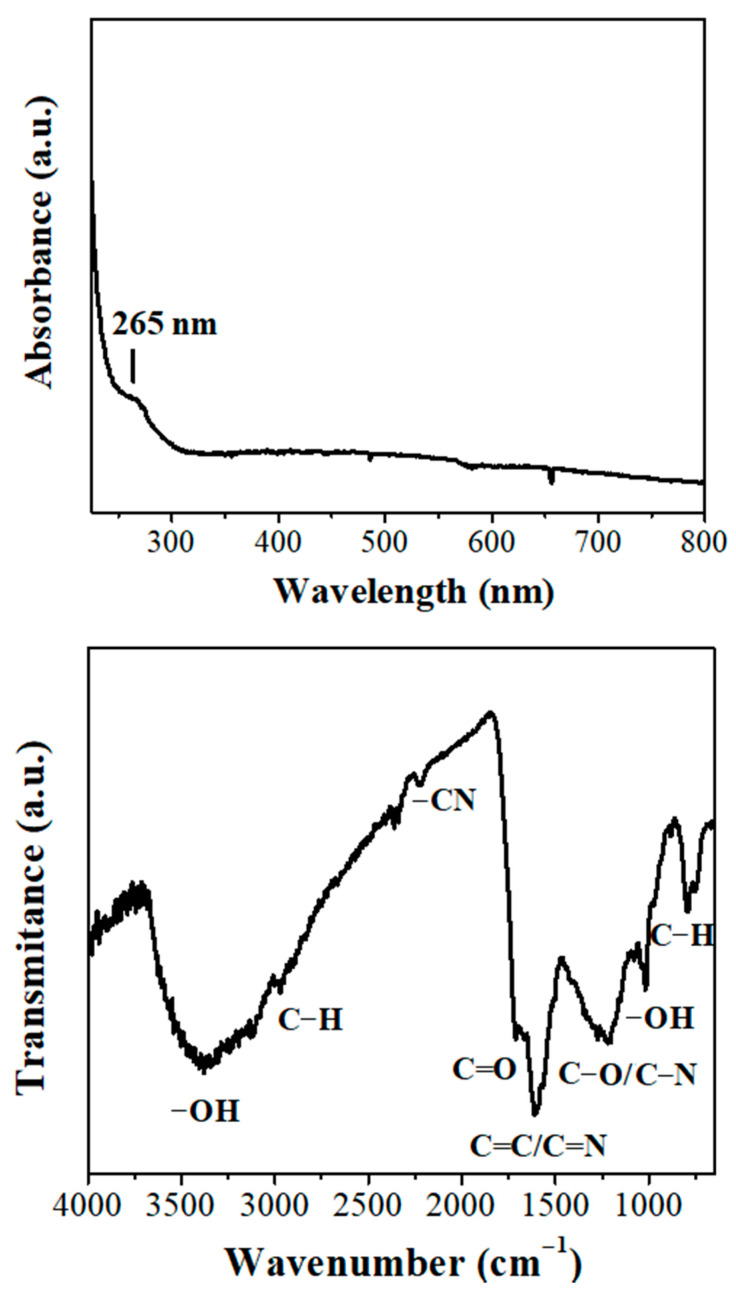
Ultraviolet (UV)-vis (**top**) and attenuated total reflectance infrared spectrum (ATR-IR) (**bottom**) spectra of carbon nanosheets.

The UV-vis spectrum of the nanosheets recorded as a fine solid suspension in ethanol exhibited a well-resolved peak at 265 nm ascribed to π–π* transitions within long-ranged sp^2^ domains (Figure 5, top). A similar absorption band has been also observed for reduced graphene oxide (270–275 nm), where chemical reduction is known to partly restore π-conjugation [27,28]. The appearance of this peak in our sample indicated a strong sp^2^ character, in line with Raman spectroscopy. The corresponding ATR infrared spectrum (Figure 5, bottom) was typical of a carbonaceous matrix (C=C/C=N 1607 cm^−1^) decorated with oxygen-containing functionalities at the surface (e.g., oxidized carbon). The strong and broad band at 3370 cm^−1^ was due to the stretching vibration of –OH. This band was accompanied by a weaker yet sharper peak at 1010 cm^−1^ assigned to the deformation mode of the hydroxyl group. Following, C–O/C–N bonds gave a broad band at 1200 cm^−1^, whereas C=O groups a discernible shoulder at 1700 cm^−1^. The material additionally contained aliphatics as evidenced by the stretching (<3000 cm^−1^) and bending (766 cm^−1^) modes of C–H group. Lastly, the small peak at 2225 cm^−1^ was ascribed to nitrile –CN.

**Figure 6:**
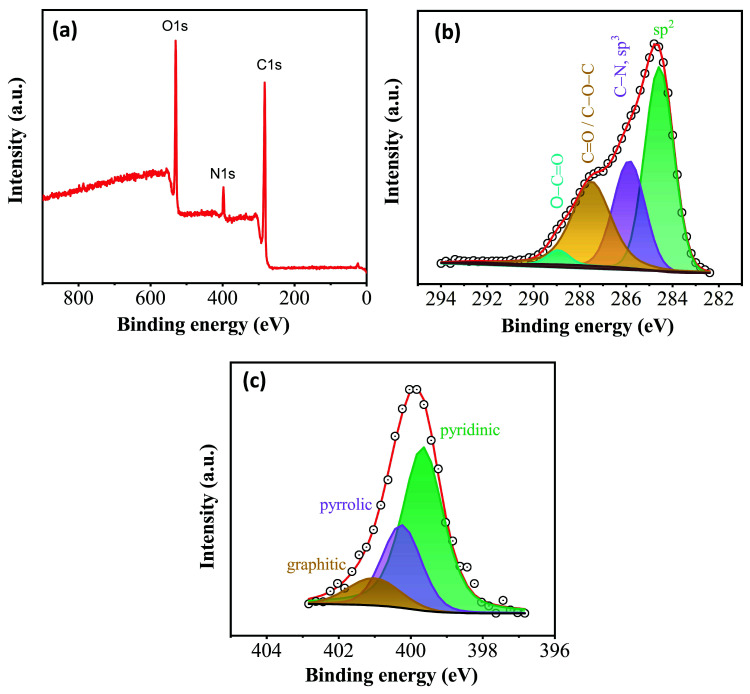
(**a**) X-ray photoelectron spectroscopy (XPS) survey spectrum of the sample. Deconvoluted high-resolution spectra of (**b**) C1s and (**c**) N1s regions.

Based on XPS spectroscopy the nanosheets contained C 70.8%, O 23.8%, and N 5.4% (Figure 6a). Nitrogen was present in the sample as a result of N-doping of the sheets by the HNO_3_ as described elsewhere [20,29]. The high-resolution spectrum for the C1s region (Figure 6b) was deconvoluted into four components corresponding to sp^2^ hybridized carbons (284.6 eV), sp^3^ and C–N type carbons (285.9 eV), as well as, carbonyl/ether (C=O/C–O–C 287.5 eV) and carboxyl groups (O–C=O, 288.9 eV) [30,31]. The deconvoluted N1s spectrum (Figure 6c) showed that nitrogen atoms were mainly present in the pyridinic configuration (399.7 eV, 62.5 at. %), followed by pyrrolic (400.3 eV, 26.6 at. %) and graphitic (401.0 eV, 10.9 at. %) types of bonding [32].

**Figure 7:**
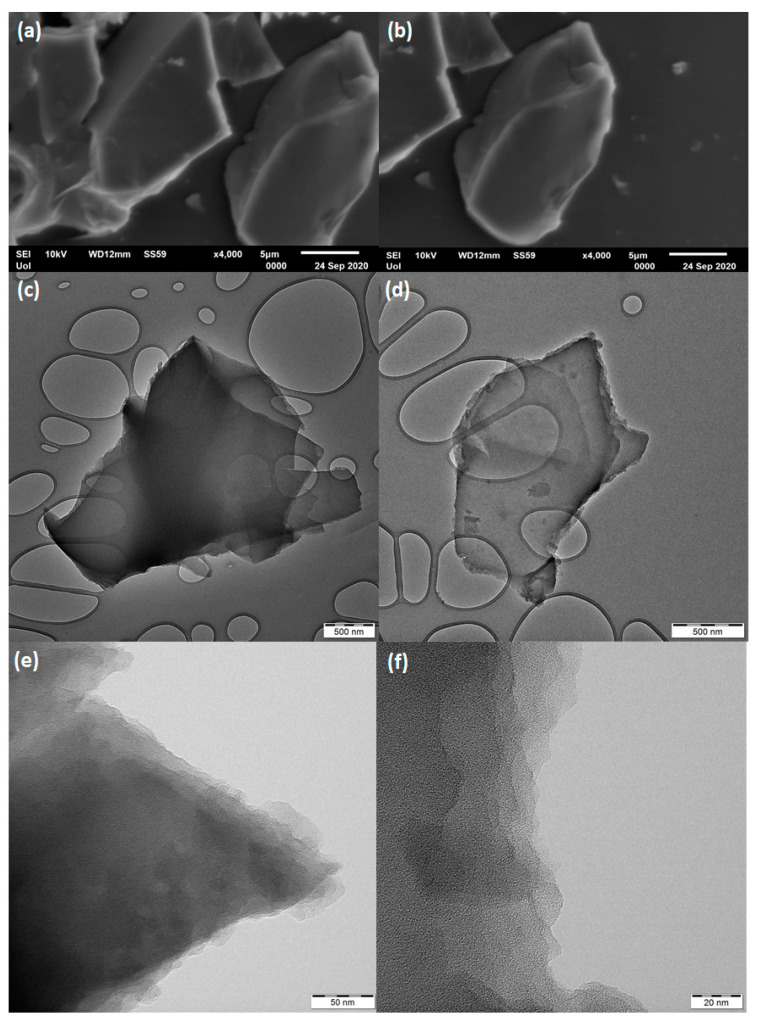
Scanning electron microscopy (SEM) (**a**,**b**) and transmission electron microscopy (TEM) (**c**–**f**) images of carbon nanosheets.

**Figure 8:**
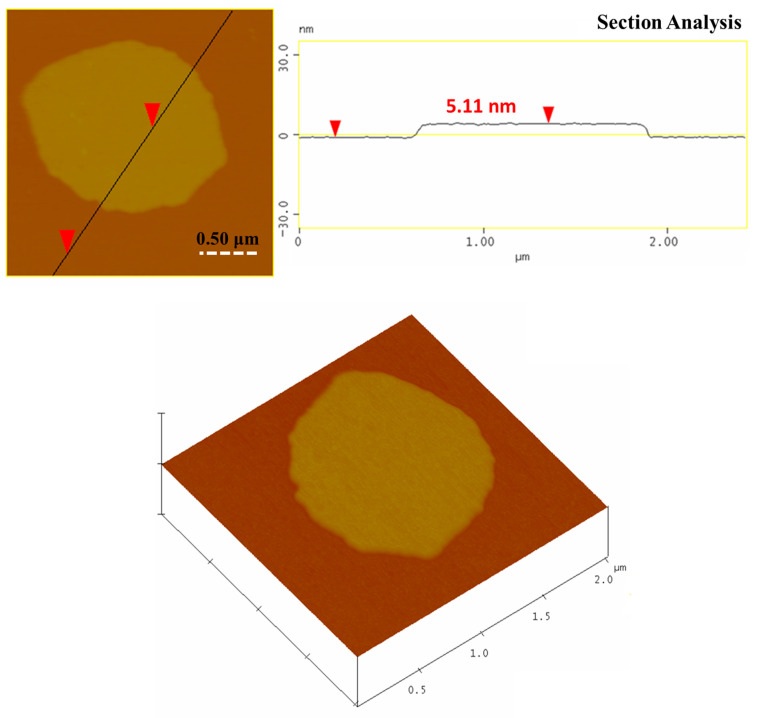
Atomic force microscopy (AFM) images of cross-sectional analysis (**top**) and 3D morphology (**bottom**) of a selected carbon nanosheet.

SEM and TEM portraits revealed exclusively the presence of carbon nanosheets in the sample (Figure 7). The nanosheets appeared compact with no surface porosity and possessing micron-sized lateral dimensions. These observations agreed well with the very low specific surface area of the sample. Furthermore, the smooth and planar surface of the sheets rather reflected the relatively high sp^2^ carbon fraction established by Raman spectroscopy [33]. Lastly, the nanosheets exhibited multi-layered texture near the edges, a feature typical of phylomorphous materials built up of stacks of individual monolayers. The average thickness of the sheets as evaluated by AFM microscopy was close to 5 nm (Figure 8). Overall, SEM, TEM, and AFM imaging confirmed the high homogeneity of the carbon solid with the appearance of nanosheets as the only nanostructure derived from the hypergolic reaction. Furthermore, no signs of impurity spots were detected on the surface of the nanosheets. 

So far, we have utilized the renowned furfuryl alcohol-fuming nitric acid rocket fuel to make carbon out of it in a one-pot synthesis. It is, however, interesting to note that there are several other bipropellant rocket fuels based on fuming nitric acid and a range of organic compounds that could equally serve this purpose. Some representative examples include alkylamines, polyamines, aromatic amines, and hydrocarbons [34,35]. However not every rocket fuel is expected to give acceptable carbon yields for practical consideration. For instance, N,N,N’,N’-tetramethylenediamine (99% Sigma-Aldrich, St. Louis, MO, USA), a green fuel used in rockets with fuming nitric acid [36], was also tested in our lab but with poor results, e.g., ignition left behind only minor carbon residue (0.2%). Still, it becomes clear that there are plenty of options for obtaining carbon from rocket fuel in the future. This is important not only from a materials synthesis point of view but also from the standpoint of managing wastes from rocket fuels or even dealing with rocket fuel disposal in a practical way. 

## 4. Conclusions

Hypergolic ignition of furfuryl alcohol by fuming nitric acid at ambient conditions led to the fast, spontaneous, and exothermic formation of carbon nanosheets in two steps: (i) Polymerization of furfuryl alcohol to poly(furfuryl alcohol), and (ii) in-situ carbonization of the polymer by an internal temperature increment near its decomposition point. The structure and morphology of the sheets were unveiled with an arsenal of techniques, such as XRD, infrared spectroscopy (Raman/IR), UV-vis, XPS, and SEM/TEM/AFM microscopies. The released energy was directly converted into useful work by either heating acetone to boiling or spinning the Crookes radiometer. In a wider sense, the furfuryl alcohol-fuming nitric acid system could form the future basis of a more general discussion about “carbon from rocket fuel,” especially considering the wealth of rocket bipropellants available today, as well as the continual progress in the field with new hypergolic fuels. Apart from its value as a new synthetic approach towards carbon materials, the present method additionally provides a way to obtain useful materials out of waste or disposed rocket propellants. Technical upgrade of the method, as well as the search of new hypergolic pairs that will provide even higher carbon yields, remains a future challenge towards safe implementation in a large scale.
